# Glycosylation end products mediate damage and apoptosis of periodontal ligament stem cells induced by the JNK-mitochondrial pathway

**DOI:** 10.18632/aging.103304

**Published:** 2020-06-30

**Authors:** Hui Fang, Kun Yang, Ping Tang, Na Zhao, Rui Ma, Xin Luo, Qi Liu

**Affiliations:** 1 Affiliated Hospital of Zunyi Medical University, Guizhou, China; 2 Zunyi Medical University, Department of Periodontology, Stomatological Hospital Zunyi, Guizhou, China; 3 Chronic Disease Control of Shenzhen, Shenzhen, Guangdong, China; 4 Third Military Medical University Daping Hospital and Research Institute of Surgery, Stomatology Chongqing, Sichuan, China; 5 Zunyi Medical University, School of Basic Medical Sciences Zunyi, Guizhou, China; 6 Department of Stomatology Pingxiang People’s Hospital Pingxiang, JiangXi, China

**Keywords:** AGEs, PDLSCs, diabetes millitus, periodontitis, oxidative stress

## Abstract

Background: Recent studies have confirmed the bidirectional relationship between the two and the exacerbation of periodontitis by type II diabetes mellitus (T2DM), the pathogenic mechanism has not yet been clarified, AGEs has been linked to the pathogenesis of both periodontitis and T2DM, JNK signaling pathway might play a important role to explain the inner mechanism.

Objectives: To study advanced glycation end products (AGEs) activate the innate immune system of the host by activating oxidative stress and affecting cellular signal transduction in periodontal ligament stem cells (PDLSCs);

Results: TNF-α and/or AGEs can induce the formation of endogenous ROS in PDLSCs, thereby activating the downstream JNK signalling pathway, leading to the initiation of the mitochondria-mediated apoptotic pathway and the induction of PDLSC apoptosis.

Conclusion: we hypothesized that the JNK pathway is a key link in the apoptosis of PDLSCs mediated by TNF-α and/or AGEs.

Materials and Methods: PDLSCs from healthy volunteers were extracted, cultured and stimulated with TNF-a and/or AGEs, Flow cytometry, CCK-8, multidifferential assay, RT-PCR, apoptosis assay, Transmission electron microscopy and Western blotting were recruit to detect the internal relations between AGEs and PDLSCs.

## INTRODUCTION

Löe has suggested that periodontitis is the sixth most common complication of diabetes mellitus (DM) [1]. Although recent studies have confirmed the bidirectional relationship between the two and the exacerbation of periodontitis by type II diabetes mellitus (T2DM), the pathogenic mechanism has not yet been clarified. The glycosylation end products (AGEs) pathway, hexosamine pathway, protein kinase C (PKC) pathway and polyol pathway are the four classical pathways through which diabetes induces complications. Michael Brownlee’s theory of the unified mechanism of diabetes complications suggests that oxidative stress may be a key factor in the abovementioned pathogenesis [2]. Recent studies have also found that oxidative stress levels in the saliva, gingival crevicular fluid and periodontal tissues of patients with T2DM with periodontitis are significantly higher than in those of patients with healthy periodontal and nondiabetic periodontitis [3–5]. Epidemiological investigations have also shown that the risk of T2DM complicated with periodontitis is extremely high and that moderate or severe periodontitis can easily develop [6]. This indicates that oxidative stress is likely to be an important factor in inducing or aggravating the destruction of periodontal tissue in diabetic patients with periodontitis. Allen et al. also suggest that the interaction between periodontitis and T2DM may occur through oxidative stress [7]. Studies have shown that AGEs produced by a chronic and persistent high glucose status in patients with T2DM can accumulate in periodontal tissues and produce excessive ROS after activation of oxidative stress. Overproduction of ROS not only directly damages periodontal tissues but also indirectly exacerbates the existing periodontal injury by indirectly promoting the release and aggregation of inflammatory cytokines (IL-1β, IL-6, IL-8, etc.) and inflammasomes (NLRP3/NALP3, etc.) and/or by activating downstream signalling pathways [8–10].

The downstream signalling pathway of AGEs can be activated by phosphorylated signalling pathway proteins. In resting cells in the physiological state, the mediators of the signal cascade are in a nonphosphorylated state, so the signalling pathway is interrupted. Once the protein upstream of the pathway is activated by phosphorylation, the entire pathway can be initiated. Jun N-terminal kinase (JNK), which is known as stress-activated protein kinase, can be activated by excess ROS to stimulate the entire pathway [11]. The JNK signalling pathway activates the proapoptotic protein Bax, inhibits the activity of the anti-apoptotic protein Bcl-2, activates c-jun/AP1 to upregulate pro-apoptotic proteins and activates P53 family proteins, thereby inducing apoptosis in different cell lines. One study found that the target of JNK transduction pathway-mediated apoptosis is mitochondria [12]. By inducing a decrease in the mitochondrial membrane potential to change the permeability of the mitochondrial membrane, the cytoplasmic small molecule solute fills the mitochondrial matrix, causing mitochondria to swell and rupture, and mitochondrial pro-apoptotic proteins, such as Cyt-c, are released into the cytoplasm. After the initiation of the caspase cascade, apoptosis is induced in the cells [13].

Under normal circumstances, periodontal tissue has a good self-renewal and repair and regeneration ability, allowing the alveolar bone and periodontal ligament to always remain in a dynamic equilibrium of reconstruction to adapt to changes in jaw size with age or changes in dietary structure to maintain periodontal support for tissue integrity and functionality. Periodontal ligament stem cells (PDLSCs) were isolated by Seo et al. in 2004 [14], and PDLSCs have since been considered to be the seed cell of choice for periodontal tissue engineering because of their potential. PDLSCs can not only differentiate into three types of periodontal tissues, namely, periodontal ligament, cementum and alveolar bone, but can also differentiate into osteoblasts, chondroblasts, adipocytes and neuroblasts by in vitro induction [15] suggesting that PDLSCs could be seed cells for renewing and repairing periodontal tissue. However, in T2DM patients with periodontitis, PDLSCs fail to exert their stem cell potential and repair damaged periodontal tissues in time. Instead, in response to the indirect effects of hyperglycaemia, periodontitis becomes more serious until the teeth are loosened and fall out. Therefore, we hypothesized that PDLSCs degenerate in T2DM patients with long-term hyperglycaemia and lose stem cell function to repair damaged periodontal tissue. In this study, we extracted normal PDLSCs from healthy people and cultured them in vitro. Based on previous research results by our group, TNF-α and AGEs were separately added to simulate environments of simple periodontitis, T2DM, and T2DM with periodontitis in vitro. Using these conditions, we explore changes in PDLSC actions and pathways to further clarify the mechanism of T2DM-induced or T2DM-aggravated periodontitis and, to some extent, to provide a new direction for the clinical treatment of T2DM with periodontitis.

## RESULTS

### Primary culture and passage of PDLSCs

**Figure 1:**
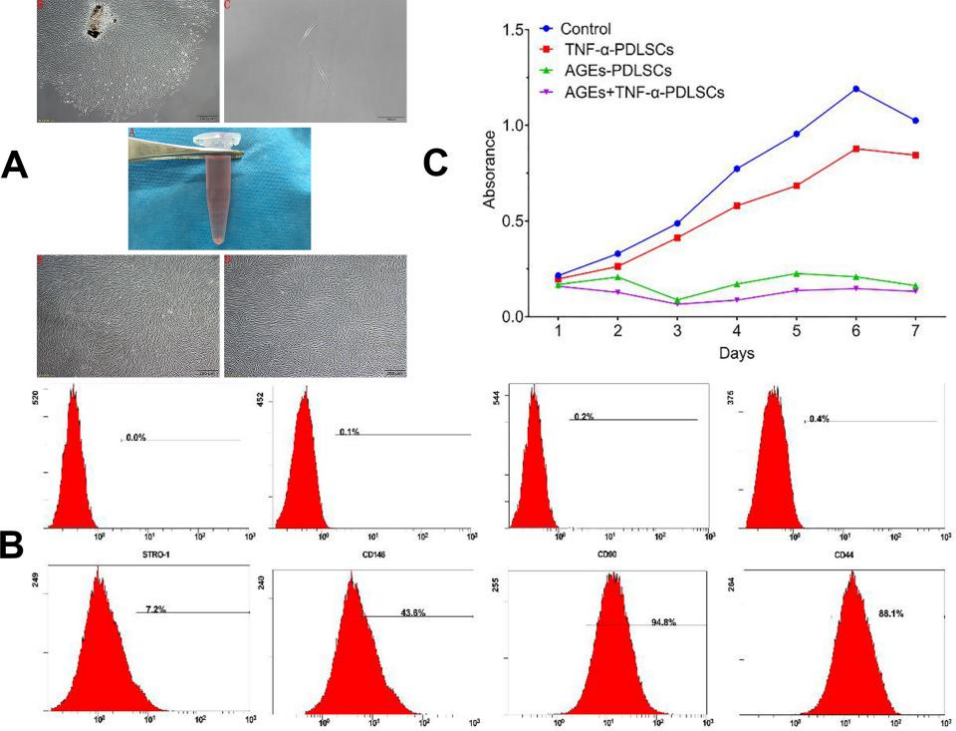
(**A**) A1 in vitro normal periodontal tissue; A2 Cells with irregular shape around the tissue block after seven days (40×); A3 primary cell monoclonal purification (40×); A4 first generation cells (40×); A5 third generation cells (40×). (**B**) PDLSCs phenotype identification PDLSCs exhibited positive expression of STRO-1, CD146, CD-90 and CD-44. (**C**) The effect of different stimuli on the proliferation of third generation PDLSCs by CCK-8 method.

In this study, primary periodontal ligament cells were extracted using the type I collagen enzymatic tissue block method. In general, cells were pulled out of the tissue around the self-tissue block on the third day. They were fusiform, long-spindle, polygonal or irregular (Figure 1A).

### Molecular phenotype of PDLSCs

**Figure 2:**
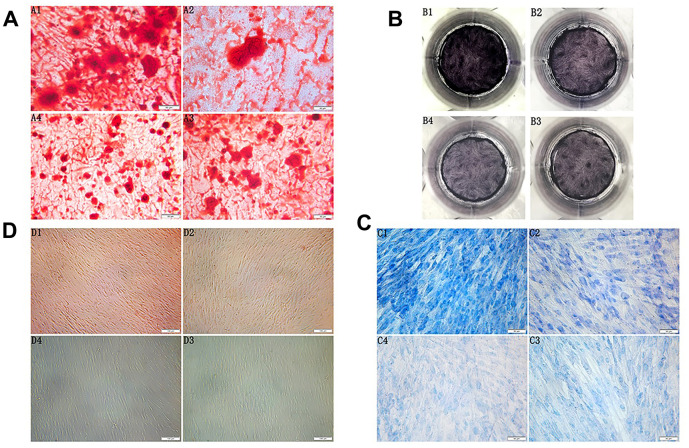
(**A**) Morphology observation of the third generation PDLSCs after twenty-one days of osteogenic induction alizarin red staining(200×) (A1: Control Group; A2: Cells were treated with 10ng/mL TNF-α; A3: Cells were treated with 100μg/mL AGEs-BSA A4: Cells were treated with 100μg/mL AGEs-BSA and 10ng/mL TNF-α). (**B**) ALP staining of the third generation PDLSCs after twenty-one days of osteogenic induction (B1: Control Group; B2: Cells were treated with 10ng/mL TNF-α; B3: Cells were treated with 100μg/mL AGEs-BSA; B4: Cells were treated with 100μg/mL AGEs-BSA and 10ng/mL TNF-α). (**C**) Morphology observation of the third generation PDLSCs after twenty-one days of chondrogenic induction toluidine blue staining(200×) (C1: Control Group; C2: Cells were treated with 10ng/mL TNF-α; C3: Cells were treated with 100μg/mL AGEs-BSA; C4: Cells were treated with 100μg/mL AGEs-BSA and 10ng/mL TNF-α). (**D**) Morphology observation of the third generation PDLSCs after 21 days of adipogenic induction oil red O staining(100×) (D1: Control Group; D2: Cells were treated with 10ng/mL TNF-α; D3: Cells were treated with 100μg/mL AGEs-BSA; D4: Cells were treated with 100μg/mL AGEs-BSA and 10ng/mL TNF-α).

The phenotypic identification of stem cells among third-generation PDLSCs was performed by flow cytometry. The results showed that the surface antigens of the third-generation PDLSCs were STRO-1 (6.97 + 0.012) %, CD146 (39.77 + 0.028) %, CD90 (93.10 + 0.009) %, and CD44 (83.27 + 0.050) % (Figure 2).

### CCK-8-based detection of the effects of different stimuli on the proliferation of PDLSCs

Compared with the control, TNF-α could promote the proliferation of PDLSCs to a certain extent, but in terms of the overall trend, TNF-α also inhibited cell proliferation. After reaching the proliferation peak on the 6^th^ day, the proliferation ability of PDLSCs began to weaken. AGEs significantly inhibited the proliferation of PDLSCs, especially on the third day. Although the inhibition was relatively weakened afterwards and the proliferative activity was increased, it was still far below the TNF-α-stimulated levels of the control group and the TNF-α-PDLSC group. The inhibition of PDLSC proliferation was strongest when AGEs and TNF-α were combined. Beginning on the first day, the proliferation of PDLSCs was significantly inhibited. The proliferation rate of the cells after two days was slower, as with the AGEs-PDLSC group. On the third day, the inhibition was the strongest, but proliferation was still slow and at a lower level. After reaching a plateau on the sixth day, the proliferation level began to slowly decline (Figure 1C).

### PDLSCs differentiated into osteoblasts, chondrocytes and adipocytes

### Osteogenic induction and alkaline phosphatase staining

After seven days of osteogenic induction, PDLSCs grew in layers, and the cell volume decreased. As the induction time increased, the local particle-like changes gradually connected into pieces, forming many scattered round-like dense mineralized nodules. The volume and the density increased, and the cells gradually transformed into osteoblasts. A large number of enlarged mineralized nodules between cells could be observed under the microscope for approximately twenty-one days. The centre was dense and dark, the surrounding contour was unclear, and the dark brown sand-like material was spread throughout the interface. The mineralized nodules of the control group were the largest of the four groups; these nodules exhibited a large number and high density and were opaque or slightly transparent. The volume of mineralized nodules in the TNF-α-PDLSC group was smaller than that in the control group, and the compactness of the nodules was weakened. The mineralized nodules in the AGEs-PDLSC group and the AGEs+TNF-α-PDLSC group were relatively minimal and dispersed, and their compactness was low. Alizarin red staining showed that each experimental group formed a single scattered mineralized nodule, namely, an orange-red precipitate (Figure 2A). The ALP staining of the PDLSCs after osteogenic induction showed that the colour of the control group was the deepest, and that of the TNF-α-PDLSC group was lighter than that of the control group. The colours of the AGEs-PDLSC group and AGEs+TNF-α-PDLSC group were significantly lighter than those of the former two groups (Figure 2B).

### Chondrogenic induction

PDLSCs were induced into chondrocytes by chondrogenesis for seven days. Cells gradually shortened, growth slowed down, the nucleus became enlarged, the nucleoplasmic ratio decreased, and cells gradually transformed into chondrocytes. After twenty-one days, cells had increased chondrocyte morphology. Toluidine blue staining showed that glycosaminoglycan was secreted in the cell matrix. In the control group, a large amount of glycosaminoglycan was secreted from the cell matrix, and the staining was the deepest of the groups; the nucleus was dark blue, and the cytoplasm was light blue. The secretion of the TNF-α-PDLSCs was lower than that of the control group, and the nuclear and cytoplasmic staining were low. The AGEs-PDLSC group and the AGEs+TNF-α-PDLSC group showed light staining. The AGEs+TNF-α-PDLSC group showed the lightest staining. Most of the cells were stained only in the nucleus and cytoplasm, and some showed no staining in either the cytoplasm or nucleus (Figure 2C).

### Adipogenic induction

After seven days of adipogenic induction of PDLSCs, most of the cells were observed to change in shape from long fusiform to short thick oval. In the control group and the TNF-α-PDLSC group, the lipid droplets gradually appeared in the cells over time. No lipid droplets were found in either the AGEs-PDLSC group or the AGEs+TNF-α-PDLSC group. After twenty-one days, oil red O staining showed that a large number of lipid droplets were formed in the cytoplasm of the control group, and a small number of lipid droplets were also observed in the TNF-α-PDLSC group. However, there were no lipid droplets in the AGEs-PDLSC and AGEs+TNF-α-PDLSC groups (Figure 2D).

### RT-PCR detection of mRNA expression of genes related to osteogenic, chondrogenic and adipogenic cell differentiation in PDLSCs

**Figure 3:**
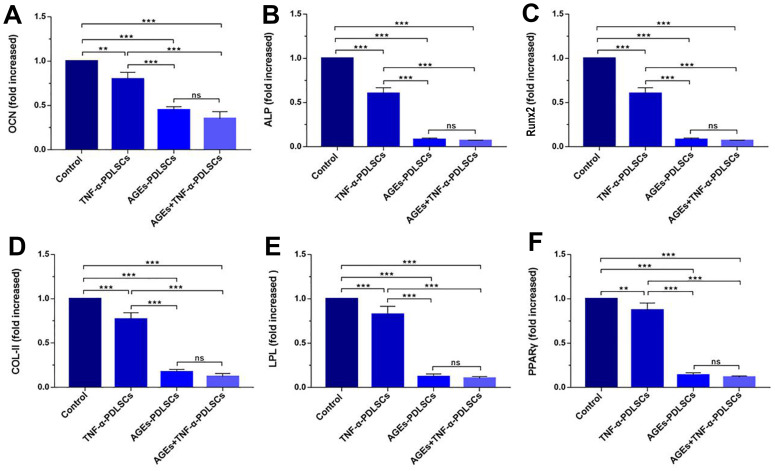
mRNA levels of OCN (**A**), ALP (**B**), RunX2 (**C**), Col-II (**D**), LPL (**E**) and PPARγ (**F**) in PDLSCs differentiated into osteoblasts, chondrocytes and adipocytes for 21 days. Data are presented as the mean±standard deviation (SD) (n=3) (ns P>0.05, * P<0.05, ** P<0.01, *** P<0.001).

Compared with the control group, the three experimental groups showed significantly reduced expression levels of the genes of interest. The reductions observed in AGEs-PDLSCs and AGEs+TNF-α-PDLSCs were more significant than those observed in TNF-α-PDLSCs. There was no significant difference between the AGEs-PDLSC group and the AGEs+TNF-α-PDLSC group (Figure 3).

### Determination of ROS, glutaraldehyde (MDA) and total mitochondrial superoxide dismutase (T-SOD) in PDLSCs

**Figure 4:**
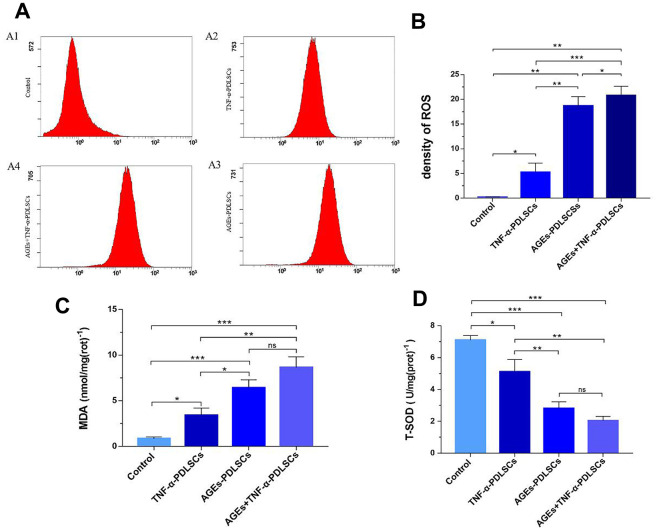
After incubating PDLSCs in four different media for 72 hours, the amount of endogenous ROS (**A**, **B**), glutaraldehyde (**C**) and total mitochondrial superoxide dismutase (**D**) produced by PDLSCs induced by AGEs and/or TNF-α was analyzed by flow cytometry (**A**). A1: Control Group; A2: Cells were treated with 10ng/mL TNF-α; A3: Cells were treated with 100μg/mL AGEs-BSA; A4: Cells were treated with 100μg/mL AGEs-BSA and 10ng/mL TNF-α. Data are presented as the mean ±standard deviation (SD) (n=3). (ns P>0.05, * P<0.05, ** P<0.01, *** P<0.001).

The ROS contents of the three experimental groups increased in turn compared with that of the control group (*P<0.05*) (Figure 4A, 4B). Malonaldehyde (MDA) can indicate the state of pro-oxidation and can reflect the degree of lipid peroxidation to a certain extent. MDA is currently recognized as one of the indicators of lipid peroxidation [16]. This experiment showed that the MDA contents of the three stimulation groups increased in turn compared with that of the control group (*P<0.05*) (Figure 4C). This is consistent with the ROS trend in each group. Superoxide dismutase (SOD) is a major antioxidant. It is divided into cytosolic copper-zinc superoxide dismutase (Cue-Zn SOD) and mitochondrial matrix-based manganese superoxide dismutase (Mn-SOD), both of which act as a bulk scavenger for superoxide and protect cells from ROS damage by scavenging superoxide radicals. Compared with the control group, the three experimental groups showed that with gradual increases in stimulation, the contents of ROS and MDA in each group gradually increased, and the level of T-SOD decreased in turn (*P<0.05*) (Figure 4D).

### Changes in cytoplasmic Ca^2+^ levels and mitochondrial membrane potential in PDLSCs

**Figure 5:**
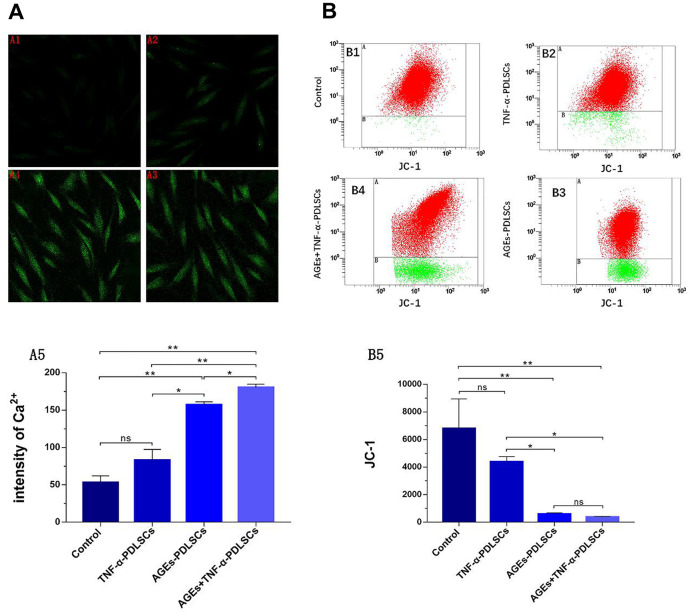
After 72 hours of PDLSCs culture in four different media, the Ca^2+^ levels of each group were detected by confocal microscopy (**A**), and the mitochondrial membrane potential (JC-1) changes of each group were analyzed by flow cytometry (**B**). Data are presented as the mean ±standard deviation (SD) (n=3). (ns P>0.05, * P<0.05, ** P<0.01, *** P<0.001).

The fluorescence intensity of the TNF-α-PDLSC group was slightly enhanced compared with that of the control group, but the difference was not statistically significant (*P>0.05*) (Figure 5A1, 5A2). The fluorescence intensity of the AGEs-PDLSC group and the AGEs+TNF-α-PDLSC group was significantly enhanced (*P<0.01*) (Figure 5A3, 5A4). Fluorescence quantitative analysis showed that the fluorescence of AGEs+ TNF-α-PDLSC group was stronger than that of AGEs-PDLSC group (*P<0.05*) (Figure 5A5).

Compared with those of the control group, the red fluorescence and green fluorescence of the TNF-α-PDLSC group decreased and increased, respectively, and the ratio of red fluorescence to green fluorescence decreased, suggesting that the depolarization ratio of mitochondrial membrane potential increased; however, the differences were not statistically significant (*P>0.05*) (Figure 5B1, 5B2). The red fluorescence of the AGEs-PDLSC group and AGEs+TNF-α-PDLSC group decreased significantly, and the green fluorescence increased remarkably (Figure 5B3, 5B4). This indicated that the mitochondrial membrane potential depolarization ratio of these two groups increased sharply, and the degree of depolarization in the AGEs+TNF-α-PDLSC group was higher than that in the AGEs-PDLSC group (*P<0.05*) (Figure 5B3, 5B4).

### Transmission electron microscopy showed changes in the mitochondrial structure of PDLSCs after seventy-two hours of different stimulation loads

**Figure 6:**
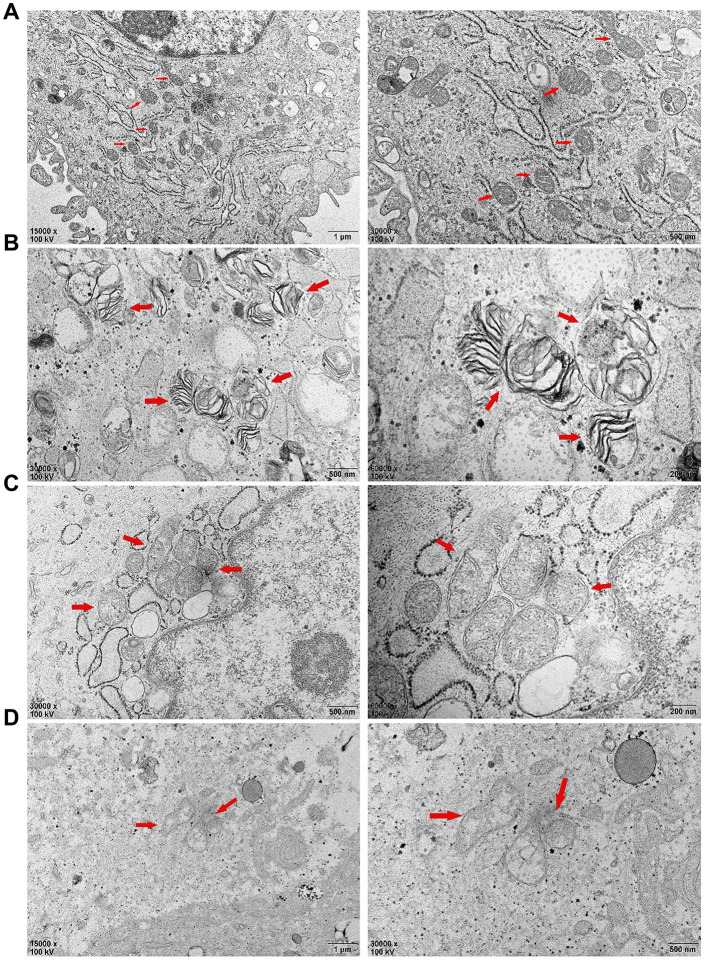
After 72 hours of PDLSCs culture in four different media, the microstructure changes of each group were observed by transmission electron microscopy: (**A**) Control Group; (**B**) Cells were treated with 10ng/mL TNF-α; (**C**) Cells were treated with 100μg/mL AGEs-BSA; (**D**) Cells were treated with 100μg/mL AGEs-BSA and 10ng/mL TNF-α.

Compared with those of the control group (Figure 6A), the mitochondrial inner and outer membranes of the TNF-α-PDLSC group disappeared; the mitochondrial ganglia were open, with a disorderly arrangement; and the substructure of the cells was basically normal (Figure 6B). A large number of cells in the AGEs-PDLSC group were in an apoptotic state, showing a high cytoplasmic concentration, cell substructure damage, organelle reduction, endoplasmic reticulum swelling, existing mitochondrial inner and outer membrane parts, the disappearance of partial destruction, open individual connections in the mitochondria ridge, individual fusion, and complete structural destruction (Figure 6C). A large quantity of cells in the AGEs+TNF-α-PDLSC group also showed an apoptotic state: the organelles were decreased, the mitochondrial membrane were gradually destroyed and dissolved, and the mitochondrial ridge was mostly destroyed and had disappeared (Figure 6D).

### Detection of PDLSC apoptosis in each group by AV/PI cell double staining

**Figure 7:**
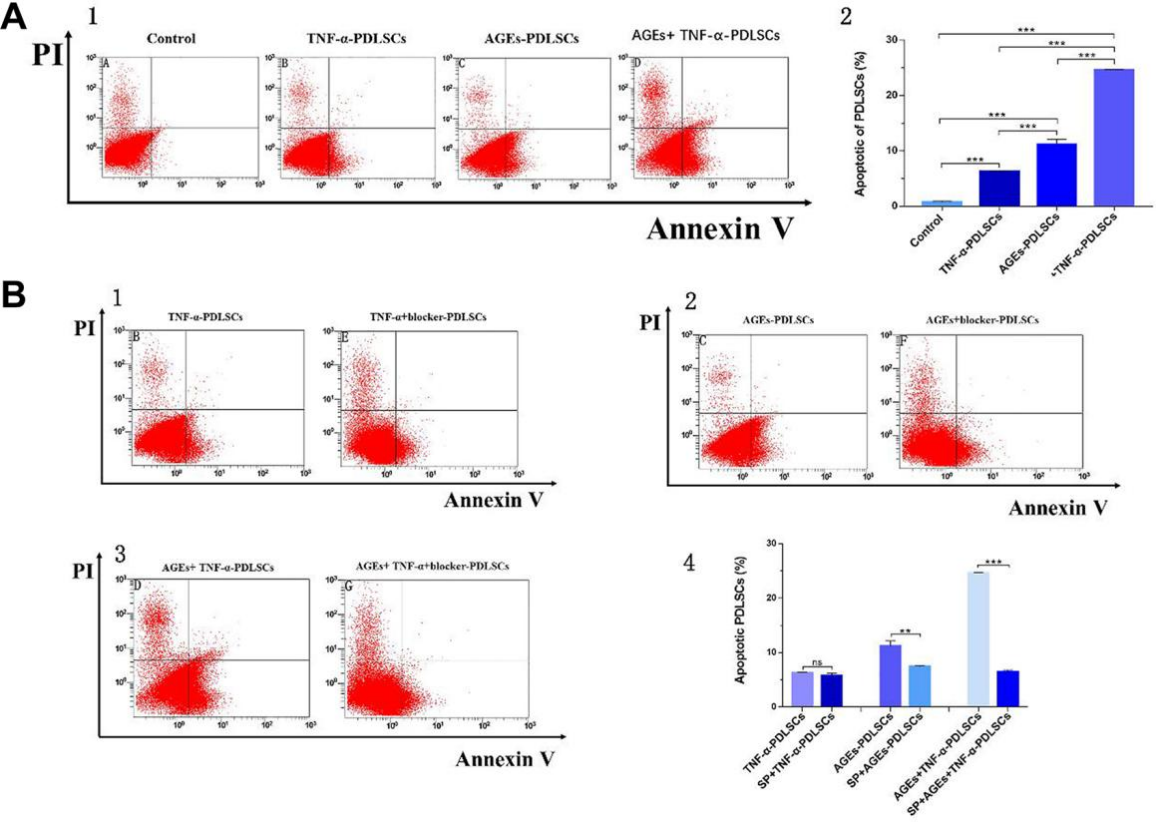
After 72 hours of PDLSCs culture in four different media, apoptosis was detected by flow cytometry detection of AV/PI double staining (**A**). Each group of cells was pretreated with the JNK pathway inhibitor SP600125 for 30 minutes and then loaded with corresponding stimuli, flow cytometry with Annexin V/PI double staining detected the apoptosis rate of each group (**B**). Data are presented as the mean±standard deviation (SD) (n=3). (ns P>0.05, * P<0.05, * * P<0.01, *** P<0.001).

Compared with that of the control group, the apoptosis rates of the three stimulation groups were significantly increased (Figure 7A). Among them, the apoptosis rate of the TNF-α-PDLSC group was lower than those of the other two stimulation groups, and the apoptosis rate of the AGEs+TNF-a-PDLSC group was the highest (Figure 7A2–7A5).

Each group of cells was pretreated with the JNK pathway inhibitor SP600125 for thirty minutes and then treated with corresponding stimuli. Compared with that of the TNF-α-PDLSC group, the apoptosis rate of the TNF-α+blocker-PDLSC group was decreased, but the difference was not statistically significant (*P>0.05*) (Figure 7B1, 7B4). The apoptosis rate of the AGEs+blocker-PDLSC group was lower than that of the AGEs-PDLSC group (*P<0.01*) (Figure 7B2, 7B4), and the rate of the AGEs+TNF-α+blocker-PDLSC group was significantly lower than that of AGEs+ TNF-α-PDLSC (*P<0.001*) (Figure 7B3, 7B4).

### RT-PCR detection of the mRNA expression of PDLSC-related genes after different stimulation treatments and SP600125 pretreatment

**Figure 8:**
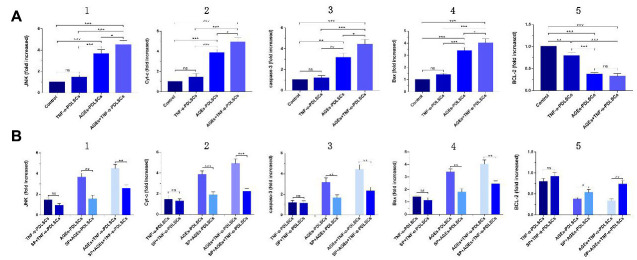
(**A**): mRNA levels and its quantification of JNK, Cyt-c, caspase-3, Bax and BCL-2 expression in different groups which were added inhibitor SP600125 before. (**B**): Each group of cells was pretreated with SP600125 for 30 minutes and then loaded with corresponding stimuli, and detect the mRNA levels and its quantification of related gene in each inhibitor group. (ns P>0.05, * P<0.05, ** P<0.01, *** P<0.001).

Compared with those of the control group, the JNK, Cyt-c, caspase-3, and Bax levels of the three experimental groups were elevated, but the differences between the TNF-α-PDLSC group and the control group were not statistically significant (*P>0.05*); the gene levels in the AGEs+TNF-α-PDLSC group were the highest of the groups (*P<0.05*) (Figure 8A1–8A4). The expression levels of BCL-2 were completely opposite to the expression levels of the above genes (Figure 8A5).

After pretreatment with SP600125 inhibitor for 30 minutes, the cells were treated with the corresponding stimulatory factor. The levels of JNK, Cyt-c, caspase-3 and Bax in each inhibitor group were decreased, but the expression of BCL-2 was increased (Figure 8B1–8B5). However, there were no significant differences between the TNF-α-PDLSC group and TNF-α+blocker-PDLSC group (*P>0.05*). These results suggest that both AGEs and TNF-α can activate the JNK pathway and induce apoptosis of PDLSCs, and when the two act synergistically, the level of activation is multiplied.

### WB detection of PDLSC-related protein expression after different stimulation treatments

**Figure 9:**
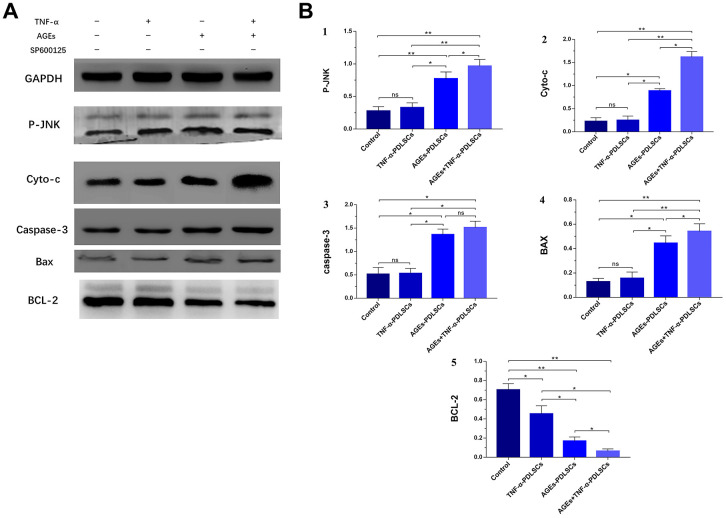
After culturing PDLSCs in AGEs and/or TNF-α medium for 72 hours, protein expression levels and its quantification of P-JNK, Cyto-c, caspase-3, Bax and BCL-2 in various experimental groups (**A**). The gray value was detected for bar graph (**B**). Data are presented as the mean±standard deviation (SD) (n=3). (ns P>0.05, * P<0.05, ** P<0.01, *** P<0.001)

The results of this experiment showed that compared with that of the control group, the expression level of the anti-apoptotic protein BCL-2 in the three stimulation groups decreased with the gradual enhancement of stimulation (*P<0.05*) (Figure 9A, 9B5), but the levels of the P-JNK, Cyt-c, caspase-3 and Bax proteins gradually increased (*P<0.05*) (Figure 9A, 9B1–9B4). However, there was no significant difference between the TNF-α-PDLSC group and the control group (*P<0.05*).

**Figure 10:**
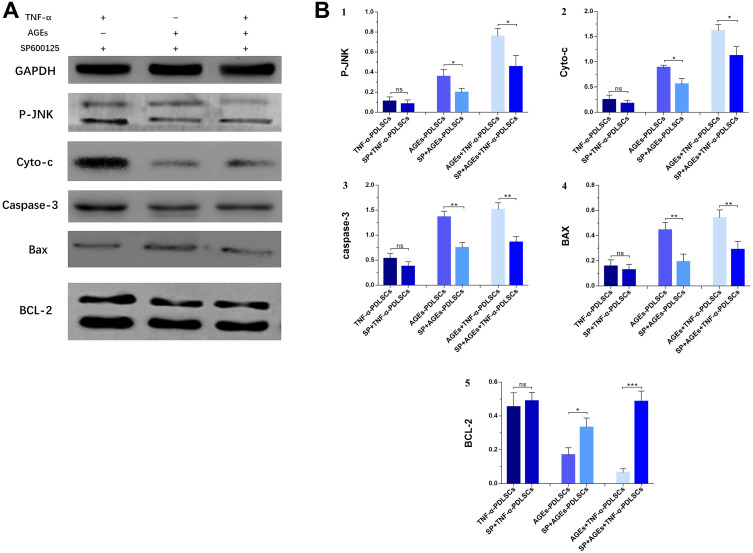
Each group of cells was pretreated with SP600125 for 30 minutes and then loaded with corresponding stimuli, protein expression levels and its quantification of P-JNK, Cyto-c, caspase-3, Bax and BCL-2 in various experimental groups (**A**). The gray value was detected for bar graph (**B**). Data are presented as the mean±standard deviation (SD) (n=3). (ns P>0.05, * P<0.05, ** P<0.01, *** P<0.001).

After pretreatment of each group of cells with SP600125 inhibitor and the corresponding stimulation, the expression of BCL-2 in each inhibitor group increased (Figure 10A, 10B5), while the levels of P-JNK, Cyt-c, caspase-3 and Bax protein decreased (Figure 10A, 10B1–10B4). However, the difference between the TNF-α+blocker-PDLSC group and the TNF-α-PDLSC group was not significant (*P>0.05*) (Figure 10A, 10B1–10B5).

## DISCUSSION

In vitro and in vivo studies have shown that a chronic inflammatory environment has a great impact on the potential of PDLSCs [17, 18]; T2DM patients with periodontitis have more severe periodontitis than patients who have simple periodontitis, and the disease progresses more rapidly and has a worse clinical treatment prognosis [18, 19]. This suggests that PDLSCs may be dysfunctional in the inflammatory environment of periodontitis and T2DM, with an attenuation or loss of ability for stem cells to regenerate. In this study, periodontal ligament cells were isolated of healthy periodontal population and stimulated with TNF-α and/or AGEs. It was found that stimulation not only affected the proliferative activity of the PDLSCs but also changed or resulted in the loss of stem cell potential. The damage to the potential of PDLSCs was even more deadly when AGEs cooperated with TNF-α (Figure 2, Figure 3). Therefore, we hypothesize that the microenvironment created by TNF-α and/or AGEs may alter the traits and/or structure of PDLSCs, leading to a decrease in and loss of function. In this study, PDLSCs were derived from the periodontal ligament, and all belonged to the same genus; however, the proliferation activity and differentiation ability of PDLSCs were greatly differed in different microenvironments. We speculated that this was due to the change in the endogenous molecular signalling mechanism in PDLSCs.

Among diabetic patients, the prevalence of periodontitis in patients with poor glycaemic control is significantly higher than that in patients with better glycaemic control. Previous studies have suggested that the tissue damage caused by periodontitis is mainly attributed to pathogenic microorganisms colonized in response to the periodontal and microbial plaque-induced host immune inflammatory response, but recent studies suggest that the tissue damage may also involve excessive release of ROS [20]. Under normal circumstances, ROS are a natural product of aerobic metabolism in the body and are eliminated by various enzymes and/or non-enzymatic systems of cellular defence to maintain the redox balance of the body. However, once ROS overproduction or clearance mechanisms are impeded, ROS can cause excessive inflammation leading to cell damage [21]. Studies have shown that the levels of AGEs and ROS in the periodontal tissues of a T2DM patient group with periodontitis are higher than those of a corresponding control group [22]; however, the antioxidant capacity (TAC, GSH, SOD) of periodontal tissue, gingival crevicular fluid and saliva decreases significantly [20]. This indicates that oxidative stress occurs in the periodontal tissues of T2DM patients with periodontitis, resulting in excessive ROS production.

Long-term hyperglycaemia in T2DM patients can induce non-enzymatic glycosylation of proteins and lipids to form heterogeneous AGEs, while the accumulation of AGEs can activate oxidative stress response [22]. Previous studies have shown that a continuous increase in AGEs may be involved in the development or facilitation of many systemic diseases and can cause a wide range of pathological effects, such as the induction of inflammatory cytokines and the enhancement of oxidative stress [23]. The results of this study show that both TNF-α and AGEs can induce oxidative stress in PDLSCs and generate endogenous ROS (Figure 4A, 4B). In addition, the oxidative stress induced by AGEs is more intense, and ROS production is higher; when AGEs act synergistically with TNF-α, oxidative stress is enhanced, and ROS production is higher than that observed with AGEs alone. The MDA and T-SOD levels in each stimulation group confirm this conclusion (Figure 4C, 4D). This is consistent with the results of clinical studies on periodontitis and T2DM with periodontitis [20]. This suggests that AGEs are potent oxidants that cause intense oxidative stress, which has an inflammatory enlargement effect, also means great damage to PDLSCs.

Apoptosis, also known as type I programmed cell death, is generally divided into two pathways: the mitochondria-mediated endogenous apoptotic pathway and the death receptor-mediated exogenous apoptotic pathway. Mitochondria-mediated apoptosis comprises crosstalk involving a complex interaction between the JNK signalling pathway and mitochondria. This interaction involves a series of signalling molecules, such as stress kinases, caspase proteases, and Bcl-2 family proteins, which release apoptotic proteins through mitochondria and initiate caspase cascade reactions to cause damage to cellular components, such as DNA, lipids, and proteins, thereby synergistically inducing apoptosis. Due to the diversity of upstream JNK pathway mediators, such as MKK4 and MK7, many different stimuli, such as cytokines, growth factors, and stress, can activate downstream factors of the JNK pathway. As a response to endogenous apoptotic stimuli, JNKs interact with different pro- or anti-apoptotic proteins of the Bcl-2 family or regulate their activities. The loss of mitochondrial membrane integrity and subsequent release of Cyt-c into the cytoplasm are important events in apoptosis. This study found that TNF-a and AGEs can induce oxidative stress and ROS formation in PDLSCs, and the persistent production and accumulation of ROS can cause great cytotoxicity to PDLSCs, resulting in activation of the JNK signalling pathway, loss of mitochondrial membrane potential, release of Cyt-c, activation of caspase-3 and upregulation of the Bax/Bcl-2 ratio. This indicates the involvement of the mitochondrial apoptotic pathway.

JNK is a member of the subfamily of mitogen-activated protein kinases (MAPKs) in mammals and is involved in cell proliferation, movement, metabolism, DNA repair and death. JNK is activated by phosphorylation in response to various cellular stresses, such as oxidative stress, osmotic shock, and inflammatory cytokines [24]. Previous studies have shown that, as a stress-activated protein kinase, JNK can be involved in the regulation of ROS-induced apoptosis after being activated by oxidative stress [24–26]. Our results indicate that both TNF-α and AGEs can cause oxidative stress in PDLSCs to produce ROS and cause apoptosis of TNF-α-PDLSCs, AGEs-PDLSCs and AGEs+TNF-α-PDLSCs (Figure 7A), and the corresponding JNK phosphorylation level is also significantly upregulated (Figure 8A1, Figure 9A, 9B1). After pretreatment of cells with the JNK-specific pathway inhibitor SP600125, the apoptosis rates of the three stimulation groups were significantly reduced (Figure 7B), and the JNK phosphorylation level was also significantly downregulated (Figure 8B1, Figure 10A, 10B1). This indicates that TNF-α and AGEs induce ROS generated by PDLSCs to activate the JNK pathway. Although this study did not explore the mechanism by which ROS induce JNK activation, previous studies have shown that ROS activate JNK primarily through two pathways: phosphorylation of JNK by ROS-sensitive apoptosis kinase 1 (ASK1) [27] and maintenance of JNK phosphorylation via inhibition of the activity of MAPK phosphatases-1 (MKP-1) in JNK inactivation [28, 29]. In addition, studies have shown that ROS can activate the JNK pathway to induce apoptosis and that JNK activation can in turn promote the formation of ROS and peroxynitrite, thereby promoting apoptosis [30]. According to Shinohara et al., phosphorylated JNK promotes intracellular ROS production by transferring fluid into the mitochondria [31]. This conclusion still needs further study for the establishment of PDLSCs.

Bax and Bcl-2 are the major regulators of the Bcl-2 protein family, and the ratio of Bax/Bcl-2 plays an important role in cell apoptosis or survival [32]. Phosphorylation activation of JNK promotes Bax translocation to the outer mitochondrial membrane, and Bax becomes a pore-forming protein of the outer mitochondrial membrane or binds to voltage-gated anion channels (VDACs). When the mitochondrial membrane permeates, the outer membrane pore becomes enlarged, and apoptosis is induced [33]. Phosphorylation of JNK induces Bad (Ser136) dephosphorylation and Bcl-2 phosphorylation and dissociates Bad from Bcl-2, resulting in increased levels of Bad and Bcl-2 [34]. Paul et al. confirmed that JNK phosphorylation promotes Bad dephosphorylation and upregulates the levels of apoptotic Bcl-2 family proteins, especially Bax and Bad, and downregulates the levels of Bcl-2 [35]. This leads to a decrease in the mitochondrial membrane potential of platelets with the release of Cyt-c and the activation of caspase, which leads to apoptosis [35]. After PDLSCs were stimulated with TNF-α and/or AGEs to produce ROS, the expression of Bax increased (Figure 8A4, Figure 9A, 9B4), while that of Bcl-2 decreased (Figure 8A5, Figure 9A, 9B5); the proportion of Bax/Bcl-2 increased with the increase in the JNK phosphorylation level (Figure 8A1, Figure 9A, 9B1). After pretreatment of cells with SP600125, the JNK phosphorylation levels of the corresponding groups of PDLSCs were downregulated (Figure 8B1, Figure 10A, 10B1). The expression of Bax was also decreased, the expression of Bcl-2 was increased, and the proportion of Bax/Bcl-2 was decreased (Figure 8B4, 8B5, Figure 10A, 10B4, 10B5). This suggests that ROS-mediated activation of JNK phosphorylation of PDLSCs can in turn induce Bax activation and Bcl-2 phosphorylation, thereby triggering apoptosis of PDLSCs.

Previous studies have confirmed that activated JNK plays an important role in ROS-mitochondria-mediated apoptosis [36]. Many key events of apoptosis are concentrated on the mitochondria, including the release of Cyt-c, changes in electron transport, loss of mitochondrial membrane potential (Δψm), and changes in cellular redox status. Almost all mitochondrial functions are related to the oxidative phosphorylation and energy coupling mechanisms located in the mitochondrial inner membrane, which consists of the intimal electron transport chain (ETC) complexes I, II, III and IV; ATP synthase; ubiquinone; and the Cyt-c composition, as electron transporters [37]. ETC is considered to be the main site of ROS production, but the mitochondrion itself is also a target of oxidative damage, resulting in impaired respiratory enzyme activity, which affects mitochondrial Δψm and further ROS production. The results showed that after PDLSCs were stimulated with TNF-a and/or AGEs, mitochondrial Δψm was significantly downregulated, and Δψm decreased more significantly in the AGEs-PDLSC group and AGEs+TNF-a-PDLSC group because of the effect of AGEs was stronger than that of TNF-a. Decreased mitochondrial Δψm is a landmark event in the early stage of apoptosis and indicates mitochondrial membrane depolarization, that membrane permeability is beginning to change, and that cells are beginning to undergo apoptosis. Therefore, the results of this study indicate that PDLSCs are stimulated by TNF-α and/or AGEs, and mitochondria-mediated apoptosis begins to occur along with the production of endogenous ROS.

The main component of mitochondria is lipid, and phospholipids account for more than 3/4 of lipids. Cardiolipin is located in the mitochondrial inner membrane as a combination of various enzymes or mosaic proteins, and Cyt-c is located on cardiolipin. Because cardiolipin is rich in unsaturated acyl chains and close to mitochondrial ETC, it becomes an early target of ROS when oxidative stress is triggered, and cardiolipin is highly susceptible to oxidative attack, causing Cyt-c to dissociate from the inner membrane. This completes the first step in the release of Cyt-c from mitochondria [38]. Oxidative damage causes oxidized cardiolipin to spread out from the mitochondrial inner membrane to the outer membrane, and the mitochondrial membrane permeability transition pore (MPTP) is formed by the release of a large amount of Ca^2+^ from the endoplasmic reticulum, Golgi apparatus and sarcoplasmic reticulum (calcium reservoir). It causes MPTP abnormality and prolonged opening, resulting in enhanced mitochondrial membrane permeability, a large number of substances having low concentrations, and potential differences in influx, leading to mitochondrial membrane depolarization, mitochondrial swelling and even mitochondrial rupture, and eventually, Cyt-c is released into the cytosol to activate apoptosis [38]. Here, TNF-α and/or AGEs stimulated PDLSCs to produce large amounts of ROS, which significantly upregulated cytosolic Ca^2+^ and Cyt-c levels in the respective groups (Figure 5A, Figure 8A2, Figure 9A, 9B2). This indicates that oxidative damage causes the Ca^2+^ transport mechanism of PDLSCs to be abnormal, resulting in a large amount of Ca^2+^ entering the cytoplasm from the calcium reservoir. Due to the enhanced permeability of the mitochondrial membrane, mitochondria overtake Ca^2+^ and cause mitochondrial Ca^2+^ accumulation, resulting in mitochondrial swelling and even lysis. This structural change can be confirmed from the observations by transmission electron microscopy in this study (Figure 6). In this study, typical characteristics of mitochondrial damage in TNF-α- and/or AGE-treated PDLSCs were observed by transmission electron microscopy, including mitochondrial swelling, double membrane decomposition, sputum lysis, and mitochondrial rupture, which confirmed that the oxidative stress induced by TNF-α and/or AGEs causes enormous, irreversible damage to PDLSCs and initiates a mitochondria-mediated endogenous apoptotic pathway to induce apoptosis of PDLSCs.

Apoptosis plays an important role in the development of multicellular organisms and in maintaining tissue homeostasis [39]. After receiving pro-apoptotic stimulation, the cells initiate and undergo apoptosis mainly via activation of caspase-2/8/9/10; then, caspase-2/8/9/10 is hydrolysed and cleaved to activate caspase-3 and caspase-7, with subsequent cleavage of a wide range of cell proteins, leading to the disintegration and apoptosis of cells [40]. The initiation of the caspase cascade indicates that the cells are entering the late stage of apoptosis, and the cells are about to become necrotic, disintegrate or become swallowed. In this study, the levels of caspase-3 in PDLSCs in the three stimulation groups increased significantly (Figure 8A3, Figure 9A, 9B3). This indicates that each group of apoptotic PDLSCs has completed early apoptosis in the mitochondrial phase, including mitochondrial Δψm downregulation, membrane permeabilization, and pro-apoptotic factor Cyt-c release, and has then formed the apoptotic complex composed of Cyt-c, apoptotic protease activator (Apaf-1), caspase-9 and ATP/dATP in the cytoplasm, which ultimately leads to the activation of the downstream effector proteins caspase-3 and caspase-7, triggering apoptosis of PDLSCs. After pretreatment of cells with SP600125, the expression of caspase-3 in the corresponding groups decreased with decreasing JNK phosphorylation levels (Figure 8B1, 8B3, Figure 10A, 10B1, 10B3). This suggests that phosphorylation activation of JNK is an upstream factor in the activation of caspse-3 and suggests that the JNK pathway is involved in the regulation of endogenous ROS-mediated apoptosis of PDLSCs.

Taken together, our results suggest that TNF-α and AGEs induce endogenous ROS produced by PDLSCs, triggering mitochondria-mediated endogenous apoptotic pathways by activating the JNK signalling pathway and inducing apoptosis of PDLSCs. This finding indicates that the chronic inflammation associated with periodontitis in patients with periodontitis alone, T2DM alone and T2DM with periodontitis can not only damage the periodontal tissues but also cause apoptosis of the PDLSCs present in the periodontal ligament. Due to persistent irreversible production of AGEs in patients with T2DM, the continuous deposition of AGEs in the periodontal tissue and the inflammatory enlargement effect of AGEs, periodontal tissue damage and the induction of PDLSC apoptosis can easily occur and/or be exacerbated, resulting in failure to repair damaged periodontal tissues. Therefore, T2DM patients are prone to periodontitis, and T2DM patients with periodontitis have severe periodontal damage and are prone to developing moderate to severe periodontitis.

## CONCLUSION

TNF-α and AGEs induce endogenous ROS produced by PDLSCs, triggering mitochondria-mediated endogenous apoptotic pathways by activating the JNK signalling pathway and inducing apoptosis of PDLSCs, The JNK pathway is a key link in the apoptosis of PDLSCs mediated by TNF-α and/or AGEs.

## MATERIALS AND METHODS

### Cell culture and materials

### Inclusion criteria

At the Department of Oral Surgery, Affiliated Stomatological Hospital of Zunyi Medical College, the premolars or third molars were selected for orthodontic treatment of fourteen-eighteen years old. Satisfy the condition: healthy teeth and periodontal tissues, previous physical health, no systemic diseases, no family history of genetics and no smoking. Informed consent was obtained from preoperative patients, which met the requirements of the Ethics Committee of the Stomatological Hospital of Zunyi Medical University.

### Extraction and cultivation of PDLSCs

Five donors with periodontal health were randomly selected, and one-third of the periodontal ligament tissue of the root was scraped, and it was digested with 0.3% type I collagenase for forty minutes at a constant temperature shaker, centrifuged at 800 r/min, and inoculated into a 6-well plate. After the cells were grown to about 80% of the area of the bottom of the well, PDLSCs were extracted by limiting dilution and cultured routinely. This study used third generation PDLSCs for testing.

### Stem cell phenotype molecular detection

The third generation of PDLSCs were packed into a 15L centrifugal tube with a density of about 3.0×10^6^ cells/mL. At room temperature, two microliters of STRO-l, CD146, CD90 and CD44 Mouse anti-human Monoclonal antibodies were added into each tube, and incubated for one hour in the refrigerator at 4 °C, then washed three times with PBS containing 3% FBS, and centrifuged at 1000 r/min for 5 min. Resuspended in PBS containing 3% FBS. The background marker was determined using the isotype control monoclonal antibody, and the fluorescent cells were analyzed by flow cytometry, and the cell surface antigen positive expression rate was calculated by a special supporting software. The unit was expressed by %.

### Detection of proliferation capacity of PDLSCs by CCK-8 method

Refer to the previous experimental results and the purpose of the experimental group (the concentration of TNF-α cannot cause a large amount of cell apoptosis), commercially available TNF-α (Pepro Tech, USA) at a concentration of 10 UG/mL was diluted to an experimental concentration of 10 ng/mL with PBS containing 1% BSA, and the experimental concentration of AGEs-BSA (Bio-Vision, USA) was 100 μg/mL. The density of the third generation PDLSCs was adjusted to 2.0×10^4^ cells/mL and inoculated into 96-well plates, four plates per plate, five wells per group. After twenty-four hours, the medium was changed to normal α-MEM (Control group), α-MEM containing 10 ng/mL TNF-α (TNF-α-PDLSCs group), α-MEM containing 100 μg/mL AGEs-BSA (AGEs-PDLSCs group), α-MEM containing 100 μg/mL AGEs-BSA and 10 ng/mL TNF-α (AGEs+TNF-α-PDLSCs group), and cultured at 37 °C in a 5% CO_2_ incubator. After twenty-four hours, a plate was randomly selected and incubated with CCK-8 solution (DOJINDO, Japan) for one hour. The absorbance was measured at 450 nm with a microplate reader (A5082-TECAN, USA) for seven days. Count the number of cells using a cell analyzer (Beckman Vi-Cell XR, USA). Calculate cell proliferation curve based on data.

### Cell staining was used to observe the differentiation of PDLSCs into osteoblasts, chondrocytes and adipocytes

The density of the third generation PDLSCs was adjusted to 2×10^5^ cells/well and inoculated into a 24-well plate. After the cells were grown to about 70%, the original culture medium was discarded, and the medium was replaced with osteogenic, chondrogenic and adipogenic induction medium (Gibco, USA). The control group was added with normal induction medium, and the other groups were added with 10 ng/mL TNF-α and/or 100 μg/mL AGEs-BSA in the induction medium according to the experimental requirements. The culture medium was changed every three days, cultured for twenty-one days, fixed with 4% paraformaldehyde for thirty minutes at room temperature. 500 ml alizarin red staining solution (osteogenic induction), toluidine blue staining solution (cartilage induction) and oil red O staining solution (fat induction) (Solarbio, China) were added to each hole and placed in incubator for twenty minutes (osteogenic and cartilage induction) and one hour respectively (fat induced), ALP staining twelve hours, discarding staining solution, PBS cleaning three times. Cartilage induction is rinsed once with absolute ethanol. Adipogenic induction wash the residual stain with 75% ethanol and 60% isopropanol. Observed under an inverted microscope and photographed. ALP staining was washed three times with PBS and photographed and compared.

### Reverse transcriptase-polymerase chain reaction (RT-PCR)

Total RNA was extracted from the third generation PDLSCs induced by osteogenic, chondrogenic and adipogenic induction medium for twenty-one days using TRIzol reagent (GIBCO, Carlsbad, CA, USA), cDNA libraries were constructed from the total RNA using a reverse transcription protocol. Expression of ALP, RUNX-2, OCN, Col-2, PPAR-γ and LPL mRNAs was analyzed using reverse transcription quantitative real-time polymerase chain reaction (RT-PCR) (Table 1).

**Table 1:** Sense and antisense primers for real-time reverse transcription-polymerase chain reaction.

**Gene**	**Sequences**
ALP	Upstream: ACTGGGGCCTGAGATACCC
	Downstream: TCGTGTTGCACTGGTTAAAGC
RUNX-2	Upstream: CCGCCTCAGTGATTTAGGGC
	Downstream: GGGTCTGTAATCTGACTCTGTCC
OCN	Upstream: AGTCCATTGTTGAGGTAGCG
	Downstream: AGACCATGCAGAGAGCGAG
Collagen type II	Upstream: TGGACGCCATGAAGGTTTTCT
	Downstream: TGGGAGCCAGATTGTCATCTC
LPL	Upstream: TCATTCCCGGAGTAGCAGAGT
	Downstream: GGCCACAAGTTTTGGCACC
PPAR-γ	Upstream: GGGATCAGCTCCGTGGATCT
	Downstream: TGCACTTTGGTACTCTTGAAGTT
GAPDH	Upstream: CACGGCAAATTCCACGGCACAGT
	Downstream: GGGGGCATCAGCAGAAGGAGCAG

### Determination of intracellular ROS, MDA and total mitochondrial superoxide dismutase (T-SOD)

Inoculate the third generation PDLSCs (3×10^6^ cells/mL) in T75 flasks. After 24 hours, it was replaced with the above four mediums and cultured continuously for 72 hours. The average level of intracellular ROS was determined using a redox sensitive dye DCFH-DA (Beyotime Biotech, Nanjing, China). Four groups of cells were washed once with PBS, 10 μmol/L DCFH-DA was incubated in an incubator at 37 °C for 20 min, washed three times with serum-free medium, and resuspended in 500 μL/tube serum-free medium by flow cytometry (FACS Calibur, Beckmen, USA). The detection of MDA and T-SOD was performed according to the manufacturer’s instructions (Jiancheng, Nanjing, China), and the absorbance values were measured at 450 nm using a microplate reader (A5082-TECAN, USA). The yield of MDA and the activity of T-SOD were calculated according to the instructions.

### Mitochondrial membrane potential (JC-1) and Ca^2+^ levels

Inoculate the third generation PDLSCs (3×10^6^ cells/mL) in T75 flasks. After twenty-four hours, it was replaced with the above four mediums and cultured continuously for seventy-two hours. Four groups of cells were stained with JC-1 dye and Fluo-4 AM at a concentration of 2 μmol/L to detect the mitochondrial membrane potential and Ca^2+^ level.

### Transmission electron microscopy to detect mitochondrial structure of each group of PDLSCs

The cells cultured in four different media for seventy-two hours were collected, and the deposited cell pellet was fixed with 2.5% glutaraldehyde solution at 4 ° C overnight, and sampled with citric acid. The ultrastructure of each group of cells was analyzed by transmission electron microscopy (TEM) (Hitachi-7500, Japan).

### Apoptosis assays

Apoptosis was measured using an Annexin V-FITC apoptosis detection kit (BD PharMingen, San Jose, CA, USA). Cells cultured in 75 cm dishes were trypsinized and collected by centrifugation. The cell pellet was washed, resuspended in 1× binding buffer and stained with annexin V-FITC as recommended by the manufacture. Cells were also stained with propidium iodide to detect necrosis. Apoptosis was analyzed by flow cytometry using a Beckman FACS Calibur.

### Reverse transcriptase-polymerase chain reaction (RT-PCR)

RNA was extracted from PDLSCs cultured in different media using TRIzol Reagent (Takara, Shiga, Japan). Total RNA was reversed transcribed into cDNA using the SYBR Premix Ex Taq kit (TaKaRa Biotechnology China). We used SYBR Green quantitative RT-PCR to determine the expression of the target genes. The primers used for this experiment were as Table 2.

**Table 2:** The primer sequences of target genes.

**Gene**	**Sequences**
JNK	Upstream: TCTGGTATGATCCTTCTGAAGCA
	Downstream: TCCTCCAAGTCCATAACTTCCTT
Cyt-c	Upstream: AAGTGTTCCCAGTGCCACA
	Downstream: ATTGGCGGCTGTGTAAGAGT
Caspase-3	Upstream: CATGGAAGCGAATCAATGGACT
	Downstream: CTGTACCAGACCGAGATGTCA
Bax	Upstream: CCCGAGAGGTCTTTTTCCGAG
	Downstream: CCAGCCCATGATGGTTCTGAT
Bcl-2	Upstream: GGTGGGGTCATGTGTGTGG
	Downstream: CGGTTCAGGTACTCAGTCATCC
GAPDH	Upstream: CACGGCAAATTCCACGGCACAGT
	Downstream: GGGGGCATCAGCAGAAGGAGCAG

### Western blot analysis

Cells in different groups were collected, washed twice with cold PBS, and lysed in RIPA lysis buffer (Beyotime, China) containing 1 mM phenylmethylsulfonyl fluoride (PMSF; Beyotime, China) and phosphatase inhibitors (Roche). Cell debris was eliminated by centrifugation at 12,000 rpm for 15 min at 4ºC. Protein concentrations were determined via Bradford protein assay. 50 μg protein per lane was loaded onto a 10% SDS-PAGE gel for electrophoresis, and then transferred onto 0.22 μm PVDF membranes (Millipore, Bedford, MA) at 300 mA for 1 h in a blotting apparatus (Bio-Rad, CA). Membranes were blocked with blocking solution (5% BSA, 0.01 M TBS and 0.1% Tween-20) at room temperature for 2 h, and subsequently incubated with primary antibodies (JNK, phosphor-JNK, Caspase-3, Bcl-2, Proteintech, China; Bax, GAPDH, Cell Signaling Technology, Inc., USA; Cyt-c, Abcam, USA) overnight at 4ºC. Finally, the membranes were rinsed with TBST (0.1% Tween-20 in 0.01 M TBS), incubated with appropriate horseradish peroxidase-conjugated secondary antibodies (1:10,000, Invitrogen, USA) at room temperature for additional 2 h, visualized by Western Blot Fluorescence Sensor (Odyssey, USA). The relative density was measured using ImageJ software.

### Statistical analysis

Data are expressed as the mean ± standard deviation. Data were analyzed by t-tests for two groups or one-way ANOVA (Tukey’s test) for multiple groups using Prism 7 software (GraphPad Software, San Diego, CA, USA). All experiments were repeated in triplicate, and P< 0.05 was indicated statistical significance.

### Ethics approval and consent to participate

Animal handling was in accordance with the Guide for the Care and Use of Laboratory Animals published by the US National Institutes of Health (NIH Publication No. 85–23, revised in 1996). This study was approved by the Animal Experiment Ethics Committee of Zunyi Medical University, Guizhou.

### Availability of data and supporting materials section

The datasets analyzed in the current study are available from the corresponding author on reasonable request.
